# Auxin/AID versus conventional knockouts: distinguishing the roles of CENP-T/W in mitotic kinetochore assembly and stability

**DOI:** 10.1098/rsob.150230

**Published:** 2016-01-20

**Authors:** Laura Wood, Daniel G. Booth, Giulia Vargiu, Shinya Ohta, Flavia deLima Alves, Kumiko Samejima, Tatsuo Fukagawa, Juri Rappsilber, William C. Earnshaw

**Affiliations:** 1 Wellcome Trust Centre for Cell Biology, Institute of Cell Biology, University of Edinburgh, Michael Swann Building, Kings Buildings, Mayfield Road, Edinburgh EH9 3JR, UK; 2 Center for Innovative and Translational Medicine, Kochi University, Kochi, Japan; 3 Graduate School of Frontier Biosciences, Osaka University, Osaka, Suita 565-0871, Japan; 4 Institute of Bioanalytics, Department of Biotechnology, Technische Universität Berlin, Berlin 13353, Germany

**Keywords:** kinetochore, CENP-T/W, chromosomes, AID system, SILAC, mass spectrometry

## Abstract

Most studies using knockout technologies to examine protein function have relied either on shutting off transcription (conventional conditional knockouts with tetracycline-regulated gene expression or gene disruption) or destroying the mature mRNA (RNAi technology). In both cases, the target protein is lost at a rate determined by its intrinsic half-life. Thus, protein levels typically fall over at least 1–3 days, and cells continue to cycle while exposed to a decreasing concentration of the protein. Here we characterise the kinetochore proteome of mitotic chromosomes isolated from a cell line in which the essential kinetochore protein CENP-T is present as an auxin-inducible degron (AID) fusion protein that is fully functional and able to support the viability of the cells. Stripping of the protein from chromosomes in early mitosis via targeted proteasomal degradation reveals the dependency of other proteins on CENP-T for their maintenance in kinetochores. We compare these results with the kinetochore proteome of conventional CENP-T/W knockouts. As the cell cycle is mostly formed from G1, S and G2 phases a gradual loss of CENP-T/W levels is more likely to reflect dependencies associated with kinetochore assembly pre-mitosis and upon entry into mitosis. Interestingly, a putative super-complex involving Rod-Zw10-zwilch (RZZ complex), Spindly, Mad1/Mad2 and CENP-E requires the function of CENP-T/W during kinetochore assembly for its stable association with the outer kinetochore, but once assembled remains associated with chromosomes after stripping of CENP-T during mitosis. This study highlights the different roles core kinetochore components may play in the assembly of kinetochores (upon entry into mitosis) versus the maintenance of specific components (during mitosis).

## 1. Introduction

The kinetochore acts as a docking site for microtubules emanating from the two spindle poles, detects improper attachments and ensures that sister chromatids are only separated after all chromosomes are bi-orientated at the metaphase plate [1–3]. The site of kinetochore assembly is specified by CENP-A, a histone H3 variant that marks the site for assembly of the constitutive centromere-associated network (CCAN), a group of 16 proteins that remains constitutively bound to centromeric chromatin throughout the cell cycle [1,4]. The CCAN links the underlying chromatin with outer kinetochore components including the KMN (KNL-1/Mis12/Ndc80) network, which can directly bind microtubules [1,5].

A complex assembled from CENP-T, CENP-W, CENP-S and CENP-X (CENP-T/W/S/X complex) forms part of the CCAN [6,7]. CENP-T/W is required for viability [6], but CENP-S/X is dispensable [8]. Despite this difference, CENP-T/W/S/X heterotetramers formed *in vitro* function together and can bind 100 bp DNA segments and induce positive supercoiling [7,9]. Close proximity of CENP-T to CENP-S has also been shown using fluorescence resonance energy transfer at centromeres [10], and supports the association of CENP-T/W and CENP-S/X heterodimers seen in structural studies [7]. Whole-chromosome proteomic studies from our laboratory indicate that kinetochores probably contain both CENP-T/W heterodimers and CENP-T/W/S/X heterotetramers [11].

In conventional silencing techniques (which include both RNAi studies and conditional gene knockouts), the speed of depletion depends on the stability of the pre-existing polypeptide. This can be problematic when trying to study proteins that function in highly dynamic cell processes such as mitosis, because cells will continue to cycle with progressively decreasing concentrations of the target protein. This can potentially select for the emergence of alternative adaptive assembly and functional pathways.

**Figure 1.**
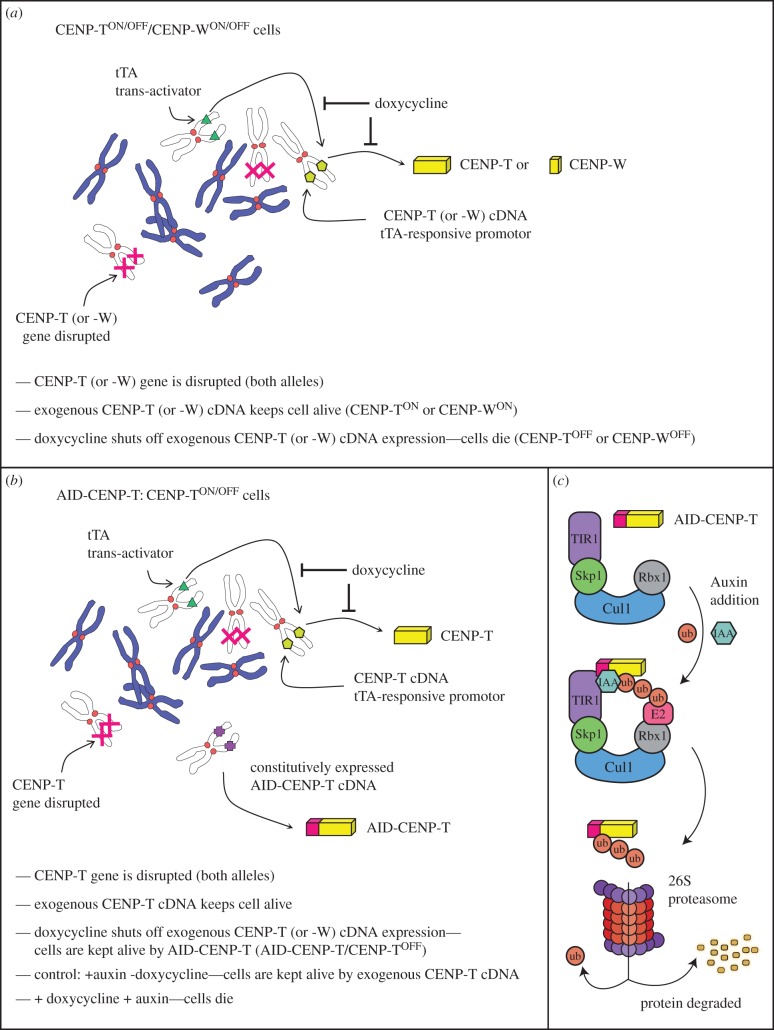
Schematic of the conditional knockout strategy and its adaption for the AID-CENP-T cell line. (*a*) Doxycycline regulated shut-down of CENP-T or CENP-W gene expression. (*b*) Combining the doxycycline regulated shut-down of gene expression with the auxin-inducible degron (AID) system. (*c*) A schematic of the auxin-induced degradation system applied in eukaryotes. The SCF E3 ubiquitin ligase consists of 4 units: Rbx1, Cul-1, Skp1 and F-box containing protein, TIR1. Auxin hormone (such as IAA) binding to the ectopically expressed TIR1 receptor promotes contacts with target proteins fused to an AID tag. The SCF-TIR1 E3 ligase can then polyubiquitinate the AID tag, promoting degradation of the substrate by the 26S proteasome.

To overcome disadvantages of conventional silencing techniques the AID (auxin-inducible degron) system was developed to create a specific, rapid and reversible method for reducing protein levels [12,13]. This system uses a plant protein degradation system in which an SCF complex containing TIR1/AFB (an auxin signalling F-box protein) promotes the degradation of Auxin/Indole-3-Acetic Acid (Aux/IAA) transcriptional repressors that regulate gene expression during development. Auxin-binding to TIR1/AFB promotes substrate-TIR1-SCF interactions, leading to substrate polyubiquitination and degradation by the proteasome (figure 1*c*) [14–16]. The core SCF components are highly conserved, but TIR1/AFB and AID-substrate orthologues do not exist in animal cells. In order to induce protein destruction, the auxin-binding region of transcriptional repressor IAA17 cloned as an AID tag was used to render target proteins sensitive to auxin addition [12].

Conditional knockouts of essential genes in chicken DT40 cells can be readily combined with the AID system [12,17,18]. Rapid degradation of CENP-H led to immediate cessation of proliferation and accumulation of DT40 cells in G2/M phase of the cell cycle [12]. Auxin-induced CENP-T degradation revealed that the Ndc80 complex is partly dependent on CENP-T for its kinetochore localization, but is also recruited by another pathway mediated by Mis12 subunits [19]. The AID system was also used in human cells, combined with siRNA-based depletion of endogenous target proteins. Using this combination, rapid removal of AID-tagged BubR1 resulted in premature mitotic exit in nocodazole-treated human cells [13].

Here, we employ quantitative mass spectrometry methods to compare the proteome of mitotic chromosomes following CENP-T depletion either by rapid auxin-induced degradation or as a consequence of normal turnover in a conditional tetracycline-repressive conditional knockout (KO) system. We confirm CENP-T's role in the recruitment of outer kinetochore proteins of the KMN. We also show that the RZZ complex, Spindly, Mad1/Mad2, CENP-E and members of the CCAN are dependent on CENP-T for their association with mitotic chromosomes only when CENP-T/W is depleted during kinetochore assembly. Our data reveal that rapid protein degradation using the AID system can distinguish dependency relationships during macromolecular complex assembly from those involved in maintaining the structure of assembled complexes in early mitosis.

## 2. Results

### 2.1. AID-CENP-T supports viability in DT40 cells

In order to test the effect of rapid CENP-T depletion on cell viability and kinetochore protein composition, we transfected CENP-T^ON/OFF^ DT40 cells [6] with a construct expressing the OsTIR1 receptor and full-length CENP-T N-terminally fused to an AID tag [12] (the cell lines used in this study are diagrammed in figure 1). In the construct used here, OsTIR1 and AID-CENP-T are encoded as one open reading frame by a single mRNA, with the two coding sequences linked by a viral T2A sequence.

**Figure 2.**
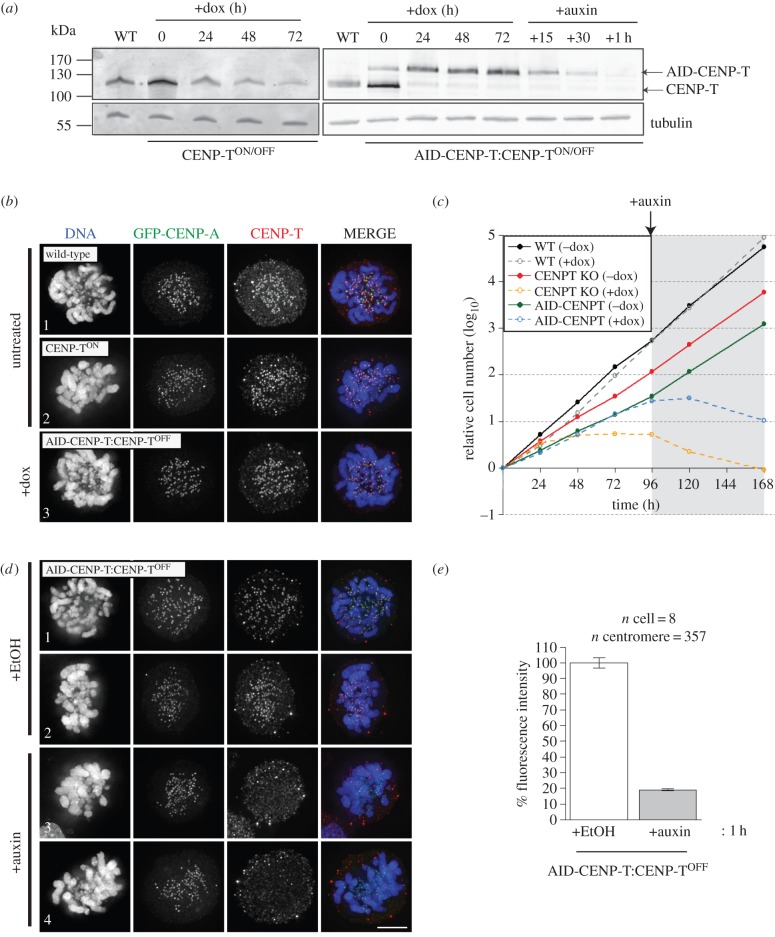
Rapid degradation of CENP-T at the protein level using the AID system. (*a*) Immunoblot detection of CENP-T in wild-type (WT), CENP-T^ON/OFF^ conditional KO or AID-CENP-T:CENP-T^ON/OFF^ cell extracts. Cell lines were treated with doxycycline for up to 3 days to turn off the expression of the CENP-T transgene. AID-CENP-T cell lines were further treated with auxin and cell lysates collected for the time points indicated. Cells (5 × 10^5^) were loaded per well and anti-tubulin blots used as a loading control. (*b*) AID-CENP-T localizes to kinetochores in doxycycline-treated AID-CENP-T:CENP-T^ON/OFF^ cells. GFP-CENP-A was expressed in wild-type, CENP-T^ON^ or AID-CENP-T:CENP-T^OFF^ cell lines and CENP-T detected using antibodies against GgCENP-T. DAPI depicts DNA staining. Scale bar: 5 µm. (*c*) Growth curves of WT, CENP-T conditional KO and the AID-CENP-T cell lines in the presence or absence of doxycycline. Doxycycline was introduced at time 0 and viable cells counted every 24 h using trypan blue staining. At 96 h, auxin was added to all cell populations and cells counted as described above. (*d*) AID-CENP-T is lost from kinetochores of doxycycline-treated AID-CENP-T:CENP-T^OFF^ cells following auxin addition. After GFP-CENP-A transfection AID-CENP-T:CENP-T^OFF^ cells were cultured with ethanol (+EtOH) or auxin (+Auxin) for 1 h. DAPI depicts DNA staining. Scale bar: 5 µm. (*e*) Fluorescence intensity measurements of GgCENP-T staining co-localizing with GFP-CENP-A loci. Approximately 20% of normal CENP-T levels remain at centromeric regions upon 1 h of auxin treatment. The graph displays the mean percentage ± s.e.m.

CENP-T is essential for cell viability. When exogenous CENP-T expression is shut off in CENP-T^ON/OFF^ cells by doxycycline addition [6], levels of the protein fall due to natural turnover and the cells lose viability within 48–72 h (figure 2*a,c*) [6]. By contrast, clones expressing AID-CENP-T (AID-CENP-T:CENP-T^ON/OFF^ cells) remained viable even after 96 h of doxycycline treatment (figure 2*c*). These cells could be maintained in doxycycline for several weeks without detectable growth defects. AID-CENP-T:CENP-T^OFF^ cell growth was unaffected by doxycycline, but the cells ceased proliferating immediately upon auxin addition (figure 2*c*).

AID-tagged CENP-T localized correctly to kinetochores (marked by transient expression of GFP-CENP-A) in AID-CENP-T:CENP-T^OFF^ cells maintained in doxycycline to silence expression of the wild-type rescuing cDNA (figure 2*b*, panel 3). CENP-T signals co-localizing with GFP-CENP-A were also clearly visible in untreated wild-type and CENP-T^ON^ cell lines (figure 2*b*, panels 1 and 2). We conclude that AID-CENP-T is functional and is able to support viability in DT40 cells.

### 2.2. AID-CENP-T is rapidly degraded during mitosis

In the CENP-T tetracycline conditional KO (CENP-T^ON/OFF^) cell line, residual levels of CENP-T were observed even after 72 h of doxycycline treatment (figure 2*a*, left panel). By contrast, after 24 h of doxycycline treatment, tetracycline-regulated CENP-T was significantly depleted in whole extracts from AID-CENP-T:CENP-T^OFF^ cells. At the same time, the levels of AID-CENP-T polypeptide rose to levels comparable with endogenous CENP-T in wild-type (WT) cells (figure 2*a*, right panel). This suggests that excess AID-CENP-T may be unstable in DT40 cells.

AID-CENP-T fusion protein levels fell rapidly within minutes of auxin addition, and no protein was detected in immunoblots after 1 h (figure 2*a*, right panel). This depletion of AID-CENP-T at mitotic kinetochores was confirmed by measuring the fluorescence intensity of CENP-T co-localizing with GFP-CENP-A. Centromeric CENP-T levels decreased by approximately 80% after 1 h of auxin treatment (figure 2*d*, panels 3 and 4; figure 2*e*). In control experiments, levels of kinetochore-associated CENP-T remained unaffected when AID-CENP-T:CENP-T^OFF^ cells were treated with ethanol (figure 2*d*, panels 1 and 2; figure 2*e*; +EtOH—auxin is dissolved in 100% ethanol).

**Figure 3.**
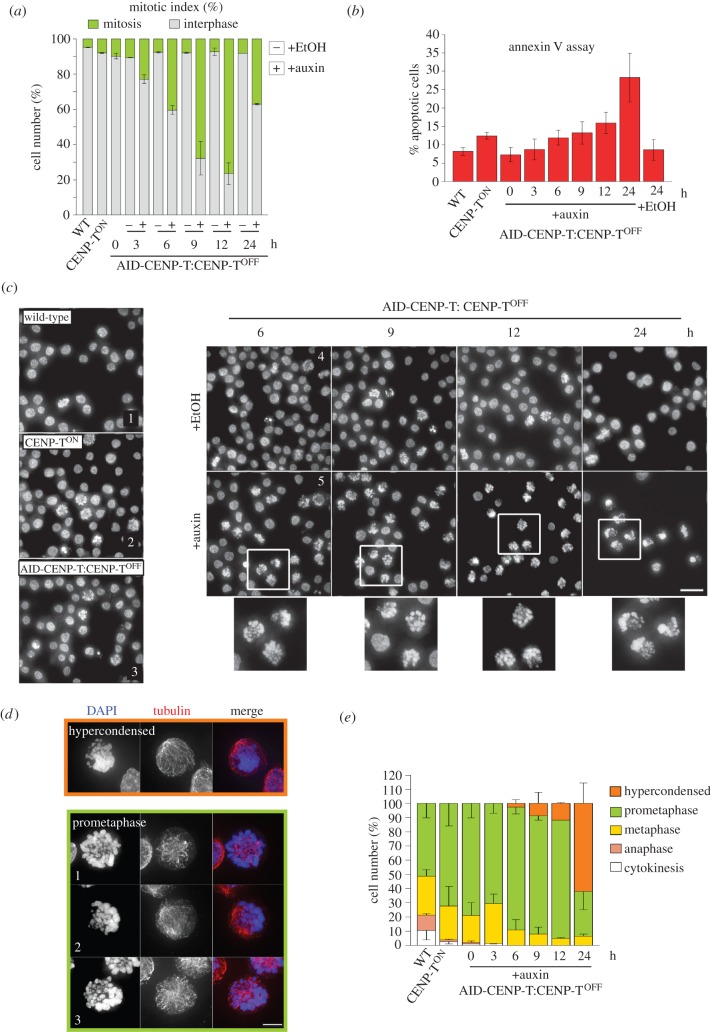
Acute degradation of CENP-T leads to prometaphase arrest and cell death. (*a*) AID-CENP-T:CENP-T^OFF^ cells were treated with ethanol (minus) or auxin (plus) for the indicated times. Graph reports the mean percentage ± s.e.m. of mitotic and interphase cell number (two repeats; *n* = ∼500 per experiment). (*b*) Fluorescence intensity of the PE-annexin V signal using flow cytometry. A graph displaying the mean percentage ± s.e.m. of apoptotic cells with high PE-annexin V signals from three repeats is shown. (*c*) Panels 1, 2 and 3 depict untreated WT, CENP-T^ON^ and AID-CENP-T:CENP-T^OFF^ cell lines, respectively. Panels 4 and 5 show AID-CENP-T:CENP-T^OFF^ treated with ethanol (+EtOH) or auxin (+auxin) for the indicated times. This was followed by fixation and staining with DAPI. The inset shows a zoomed in view as defined by the white squares. Scale bar: 20 µm. (*d*) Images showing phenotypes scored in panel (*e*). Hypercondensed: chromosomes that have a severe hypercondensed appearance. Prometaphase 1: typical prometaphase seen in a control cell. Prometaphase 2 and 3: prometaphase morphologies commonly observed in auxin-treated cell populations. Scale bar: 5 µm. (*e*) Scoring of mitotic phenotypes seen after auxin addition. After the indicated time period, cells were processed for immunofluorescence and stained with DAPI (blue) and anti-tubulin antibodies (red), and categorized into mitotic phases and phenotypes highlighted in (*d*). The means as a percentage ± s.e.m. are depicted for two repeats (*n* = ∼100 per experiment). Cells were categorized into mitotic phases and phenotypes highlighted in (*d*).

Rapid degradation of CENP-T using the AID system led to a cell cycle arrest with 76% of cells blocked in mitosis following 12 h of auxin treatment (figure 3*a*). After 24 h of auxin addition the mitotic population of AID-CENP-T:CENP-T^OFF^ cells fell to 37% (figure 3*a*). This was explained by an increase in apoptotic index observed using the annexin V assay (figure 3*b*). These observations suggest that cells initially arrest in mitosis and ultimately progress to cell death.

AID-CENP-T:CENP-T^OFF^ cells initially accumulated in prometaphase upon treatment with auxin (figure 3*c*). When compared with typical prometaphase cells (figure 3*d*, panel 1) auxin-treated cell populations often exhibited a large cluster of chromosomes displaced to one side of the cell (figure 3*d*, panels 2 and 3). Hypercondensed chromosomes were seen in approximately 62% of the mitotic population after 24 h of auxin treatment (figure 3*d*,*e*—‘Hypercondensed’). This phenotype was more severe than that observed in conventional CENP-T^OFF^ cells [6].

**Figure 4.**
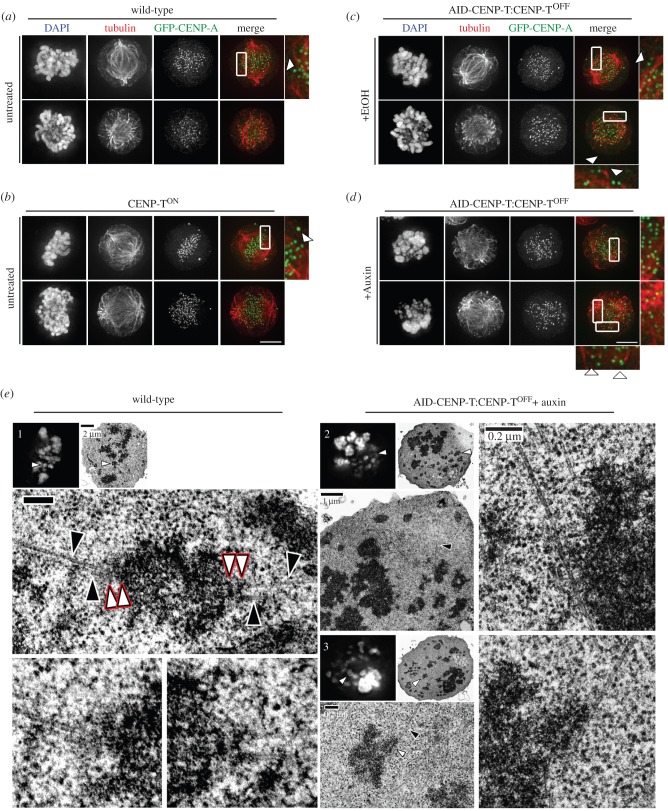
CENP-T loss leads to microtubule attachment defects. GFP-CENP-A was transfected into (*a*) wild-type, (*b*) CENP-T^ON/OFF^ conditional knockouts and (*c*,*d*) AID-CENP-T:CENP-T^ON/OFF^ cells. Attachment to PLL-coated coverslips was followed by fixation and staining using antibodies against tubulin. DAPI depicts DNA staining. Scale bar: 5 µm. AID-CENP-T:CENP-T^OFF^ cells were either cultured with (*c*) ethanol (+EtOH) or (*d*) auxin (+auxin) for 6.5 h. (*e*) (1) Light microscopy image (DAPI; left) of a wild-type metaphase cell subsequently processed for thin-section transmission electron microscopy (TEM; right). White arrows depict a metaphase aligned chromosome, with lower panels displaying magnified electron micrographs of the chromosome of interest. In these images red rimmed arrows highlight inner and outer kinetochore plates, while black arrows signify microtubules. (2) Mild and (3) severe phenotypes associated with rapid AID-CENP-T degradation. AID-CENP-T cells were treated with auxin for 6.5 h before immediate processing for electron microscopy. Cells analysed by light microscopy (DAPI; left) and TEM (right) are depicted. White arrows mark the chromosome of interest. Magnified EM images show sites of potential lateral microtubule interactions. Black arrows depict microtubules, while white arrows show possible interactions with chromosomes. Scale bars as indicated.

To complete prometaphase chromosome alignment, kinetochores must form bipolar attachments with opposing spindle microtubules. In untreated wild-type populations (figure 4*a*) and CENP-T^ON^ cells (figure 4*b*) bioriented attachments were readily observed (arrowheads). AID-CENP-T:CENP-T^OFF^ cells treated with auxin displayed problems in forming bioriented kinetochore-microtubule attachments following CENP-T degradation when compared with the ethanol-treated controls (figure 4*c*,*d*). After 6.5 h of auxin treatment only a few CENP-A signals appeared to form end-on associations with spindle microtubules (figure 4*d*, arrowheads, lower panel).

This loss of end-on microtubule attachments was confirmed by correlative light and electron microscopy (CLEM) (figure 4*e*). In wild-type cells, we could find kinetochores attached end-on to spindle microtubules emanating from opposite poles of the cell (figure 4*e*, panel 1). No such attachments were detected in cells treated with auxin for 6.5 h. More commonly, chromosomes in CENP-T-depleted prometaphase arrested cells appeared to remain unattached or possibly formed lateral associations with the microtubules (figure 4*e*, panels 2 and 3).

We conclude that AID-CENP-T incorporation at centromeres does not affect its ability to be rapidly degraded by the ubiquitin ligase system and rapid destruction of CENP-T compromises the ability of chromosomes to form normal end-on attachments to microtubules.

### 2.3. Chromosomal AID-CENP-T levels do not recover following degradation in mitotic cells

**Figure 5.**
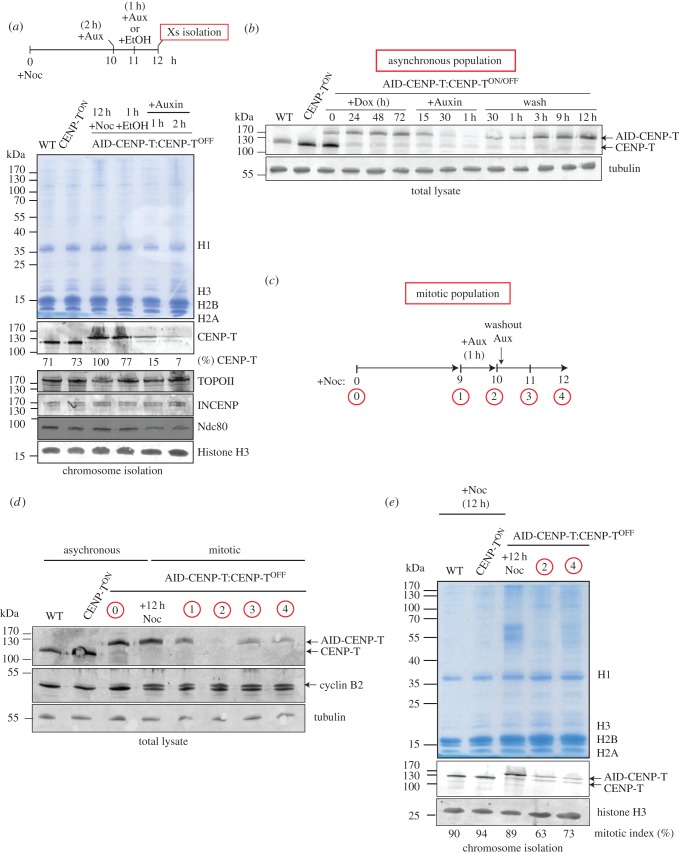
AID-CENP-T protein levels are not reversible in mitosis. (*a*) Schematic of indicated lengths of nocodazole and auxin treatments before chromosome isolation procedures. Coomassie stained gel showing the chromosome isolates from wild-type (WT), CENP-T^ON^ and AID-CENP-T:CENP-T^OFF^ cell lines. Chromosomes isolated from the AID-CENP-T cell line after ethanol (+EtOH) or auxin treatment (+Aux) for the indicated time periods are also shown. Corresponding immunoblots of chromosome samples using anti-GgCENP-T, anti-GgINCENP, anti-GgTopo IIalpha, anti-GgNdc80 and anti-Histone H3 antibodies are depicted. (*b*) Immunoblot detection of CENP-T and tubulin in whole-cell extracts. Doxycycline was added at time 0 and cells harvested every 24 h for 3 days. AID-CENP-T:CENP-T^OFF^ cells were further treated with auxin and cell lysates taken 15 min, 30 min and 1 h after addition. This was followed by additional wash steps to remove the auxin and cell lysates taken as indicated. Cells (2.5 × 10^5^) were loaded per well. (*c*) Schematic of nocodazole and auxin treatments to test whether auxin-induced degradation of AID-CENP-T was reversible in mitosis. (*d*) Western blot analysis of whole-cell extracts from asynchronous and synchronised mitotic cell populations (as depicted in 5*c*). Blots were probed with antibodies against GgCENP-T, GgCyclinB2 and tubulin. (*e*) Coomassie stained gel of chromosomes isolated under the indicated conditions. Immunoblots of corresponding chromosome isolations using anti-GgCENP-T and anti-Histone H3 antibodies. The indicated mitotic index (%) of the individual cell populations at the time of chromosome isolation procedures is depicted.

We next wished to confirm the extent to which the rapid degradation of CENP-T from AID-CENP-T:CENP-T^ON/OFF^ cells was reversible upon the removal of auxin. In order to measure CENP-T levels on mitotic chromosomes, cells blocked in mitosis using a 12 h treatment with nocodazole (+Noc) were exposed to either auxin (+Aux) or ethanol (+EtOH) according to the protocol diagrammed in figure 5*a*, and CENP-T levels were measured by quantitative immunoblotting. Levels of AID-CENP-T on mitotic chromosomes fell to 15% and 7% of the starting levels in cells treated with auxin for 1 or 2 h, respectively (figure 5*a*). In controls, DNA topoisomerase IIalpha and INCENP levels did not change significantly upon CENP-T degradation, although Ndc80 levels appeared to be reduced.

AID-CENP-T degradation is reversible in asynchronous populations of AID-CENP-T:CENP-T^OFF^ cells. AID-CENP-T levels were restored within 30 min after washing out the auxin (figure 5*b*). To ask whether a similar recovery could be observed in mitotic populations, CENP-T levels in nocodazole-arrested auxin-treated AID-CENP-T:CENP-T^OFF^ cells were examined in whole-cell extracts (figure 5*d*) and isolated chromosomes (figure 5*e*) before and after a 1–2 h washout of the auxin as outlined in figure 5*c*. In total lysates, AID-CENP-T signals were absent after 1 h of auxin treatment, but partially recovered following auxin removal (figure 5*d*). Levels of mitotic chromosome-associated AID-CENP-T were also significantly reduced after 1 h of auxin treatment, but failed to return even 2 h after an auxin washout (figure 5*e*).

These experiments show that the AID system works efficiently in both mitotic cells and asynchronous cultures. CLIP-tagged CENP-T, CENP-W, CENP-S and CENP-X have been shown to load onto centromeres in late S phase and G2 [10,20], and the lack of CENP-T recovery on chromosomes could be explained if AID-CENP-T is unable to reload onto chromosomes in mitosis. However, live cell imaging has shown that GFP-CENP-T levels at kinetochores significantly increase immediately following NEBD [21] and we thus believe it to be more likely that AID-CENP-T levels do not recover in mitotic cells due to the inhibition of CENP-T protein synthesis. The rapid recovery of CENP-T levels in asynchronous cells following auxin removal is likely to be confined to the interphase population.

### 2.4. Quantitative proteomics reveals different dependencies associated with rapid CENP-T depletion versus slow CENP-T or CENP-W depletion

**Figure 6.**
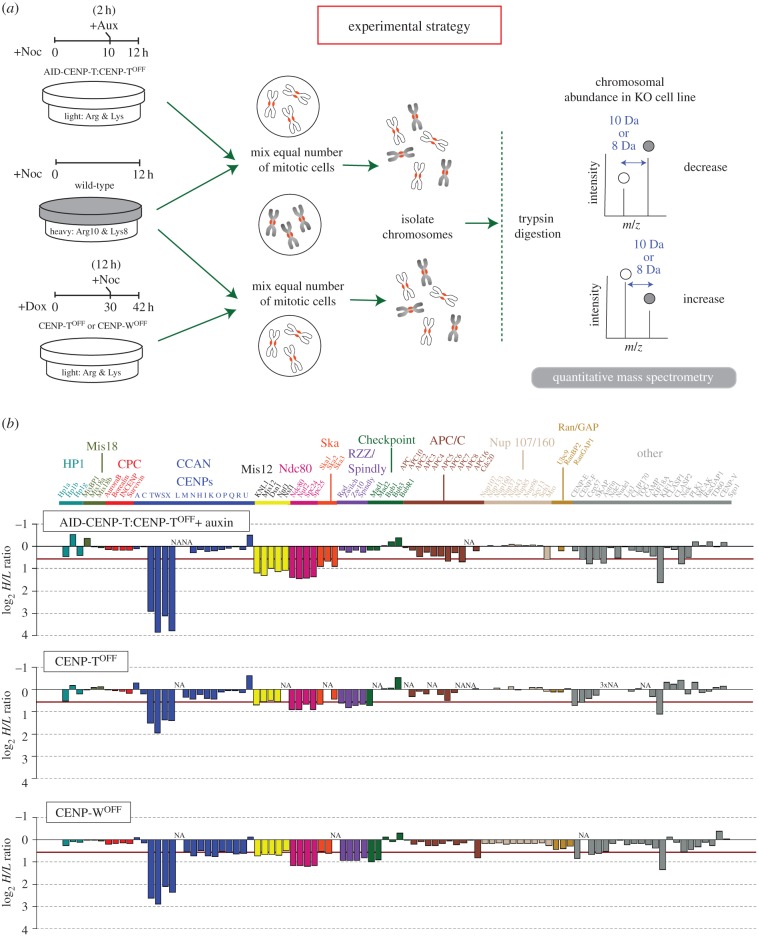
Identification of CENP-T-dependent chromosomal proteins using proteomics analysis. (*a*) Schematic of the SILAC/proteomics experimental strategy. Wild-type cells are grown in heavy SILAC medium, while the AID-CENP-T:CENP-T^OFF^ cells are cultured in the presence of light amino acids. AID-CENP-T cells are maintained in doxycycline to ‘turn off’ the expression of the inducible transgene and ensure that AID-tagged CENP-T becomes the dominant form of the protein. Both cell types are blocked in mitosis with nocodazole (+Noc) for 12 h. Ten hours into the nocodazole block auxin is added (+Aux) to the AID-CENP-T:CENP-T^OFF^ cells to promote the degradation of the AID-tagged protein. The CENP-T and CENP-W KO cell line is cultured in the presence of light amino acids and doxycycline (+Dox) added for 30 h before nocodazole addition. Equal numbers of mitotic cells from the two populations are mixed and the chromosomes isolated. After chromosome isolation, mass spectrometry is used to determine SILAC ratios for individual proteins. (*b*) Barplots of mean log_2_ *H*/*L* ratios from AID-CENP-T:CENP-T^OFF^, CENP-T^OFF^ and CENP-W^OFF^ SILAC experiments. The red line indicates a greater than 1.5-fold-change in the abundance of a protein. All assemblies listed have previously been identified as centromeric, kinetochore components or proteins closely associated with kinetochore-fibres (K-fibres). Groupings have been made based on the current literature of kinetochore subcomplex formation.

Mass spectrometry was used to analyse differences in the abundance of the total kinetochore proteome on isolated mitotic chromosomes, comparing rapid degradation of AID-CENP-T from auxin-treated AID-CENP-T: CENP-T^OFF^ cells with CENP-T (or CENP-W) loss over multiple cell cycles in conventional CENP-T^OFF^ or CENP-W^OFF^ conditional knockouts. SILAC-based proteomics was performed according to the protocol shown in figure 6*a*. Auxin was used to deplete AID-CENP-T, while doxycycline was added to turn off expression of the rescuing cDNA in CENP-T^OFF^ or CENP-W^OFF^ cells.

In this analysis, we were able to reproducibly quantify 86–96 kinetochore-associated proteins with highly correlative H/L SILAC ratios between biological replicates (*R* = 0.839–0.920; electronic supplementary material, figure S1). Outliers were excluded if the fold difference in SILAC ratios between biological replicates for a given protein was greater than ±2 s.d. away from the mean. For example, CENP-M, APC8 and CENP-F were not considered in AID-CENP-T:CENP-T^OFF^, CENP-T^OFF^ and CENP-W^OFF^ proteomics analysis, respectively (electronic supplementary material, figure S1). A list of all identified kinetochore proteins and their assigned SILAC ratios after normalization to Histone H4 ratios can be found in the electronic supplementary material, table S1.

CENP-X is a small (80 aa) protein formed mostly from a histone-fold like domain [7,8] that has a low number of unique peptide sequences available for quantitation. Because MaxQuant assigned CENP-X a SILAC ratio in only a single AID-CENP-T:CENP-T^OFF^ proteomics experiment special procedures were used to quantitate this protein. To validate H/L SILAC ratios, we identified a unique CENP-X peptide that could be successfully used for quantitation across both AID-CENP-T proteomics samples using Skyline (for details see Material and methods, and electronic supplementary material, figure S2*a*–*c*) [22,23].

**Figure 7.**
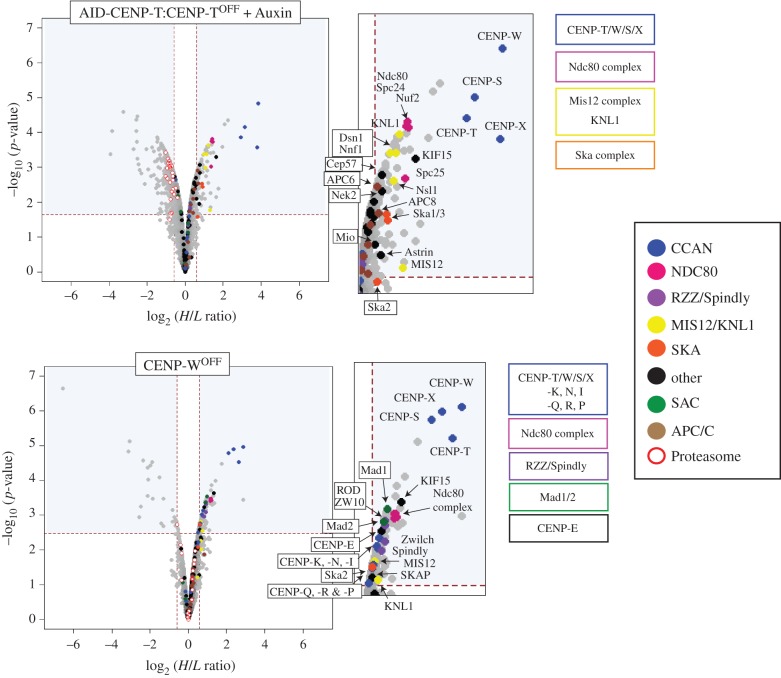
Comparison of significantly depleted chromosomal proteins after conditional shut-down or auxin-induced degradation of CENP-T. Volcano plots represent the average log_2_ fold-change between two biological replicates versus –log_10_ (*p-*values) calculated using the limma package in R [24–27]. Proteins that have an adjusted *p* < 0.05 and a H/L SILAC ratio representative of a greater than 1.5-fold-change are found within the significant areas indicated by the blue boxes. Key kinetochore proteins are colour coded based on known subcomplex formation.

In our proteomics experiments downward bars in figure 6*b* show depleted proteins, whereas upwards bars show proteins whose levels on chromosomes increased. All proteins depicted have been shown to bind centromeres, form core kinetochore complexes or closely associate with kinetochore-fibres (K-fibres). We found that CENP-T levels were only two- to threefold reduced in chromosomes isolated from CENP-T^OFF^ cells at 42–43 h after doxycycline addition (figure 6*b*; electronic supplementary material, table S1, figure S3*a*). This time point was chosen as the longest doxycycline treatment compatible with cultures achieving a sufficient mitotic index to allow efficient mitotic chromosome isolation. Within the same time frame a more complete depletion of CENP-T on chromosomes isolated from CENP-W^OFF^ cells was shown by proteomics analysis (figure 6*b*; electronic supplementary material, table S1) and immunoblotting (electronic supplementary material, figure S3*a*); for this reason statistical analysis of downstream dependencies using the inducible KO system was performed using CENP-W^OFF^ cells only (figure 7).

To identify proteins whose associations with mitotic chromosomes showed statistically significant differences after loss of CENP-T, *p*-values and adjusted *p*-values were calculated using the limma package in Bioconductor [24–27], producing the ‘volcano’ plots shown in figure 7. Proteins within the upper right quadrant and upper left quadrant represent proteins that have H/L SILAC ratios representing a greater than 1.5-fold-change (and adjusted *p* < 0.05). Those to the left are increased, whereas those to the right are decreased. All proteins significantly depleted from AID-CENP-T:CENP-T^OFF^ or CENP-W^OFF^ chromosomes are highlighted in the electronic supplementary material, table S2.

As expected, rapid degradation of AID-CENP-T led to a substantial depletion of CENP-W, CENP-S and CENP-X from isolated mitotic chromosomes, confirming that these proteins form a highly inter-dependent complex [7] (figure 7). A similar result was seen in conditional KO cell lines, although depletion of the CENP-T/W/S/X complex was only partial in CENP-T^OFF^ chromosomes (described earlier) and less effective in CENP-W^OFF^ cells (figure 6*b*).

This difference in the loss of CENP-T levels could also be seen in chromosome spreads comparing wild-type, CENP-W^OFF^ and AID-CENP-T:CENP-T^OFF^ cells subjected to the same drug treatments as outlined in figure 6*a* (electronic supplementary material, figure S3*b*,*c*). Although CENP-T signals at the primary constriction sites were reduced in both cell lines when compared with wild-type controls, AID-CENP-T depletion was more extensive. By contrast, CENP-A levels at the primary constriction sites remained the same across all cell lines (electronic supplementary material, figure S3*b*,*c*).

In addition to members of the CENP-T/W/S/X complex, several outer kinetochore complexes were also affected. Levels of chromosome associated KMN and Ska complex were significantly reduced in AID-CENP-T:CENP-T^OFF^ chromosomes (figure 7; Ska2 lies just outside this boundary). Although Ndc80 complex components were also depleted from chromosomes isolated from CENP-T^OFF^ and CENP-W^OFF^ cells, in comparison a smaller reduction of Mis12 and Ska complex components was observed (figure 6*b*). Only Mis12, KNL-1 and Ska2 are significantly depleted in CENP-W^OFF^ chromosomes (figure 7). The more effective depletion of CENP-T/W/S/X in AID-CENP-T proteomics experiments may account for this difference. Interestingly, loss of the CENP-T/W/S/X complex leads to only a partial loss of KMN components. CENP-C is unaffected by CENP-T depletion (figure 6*b*; electronic supplementary material table S1) and, as suggested by other studies, may support a separate path for KMN recruitment [19,28–33]. However, there is abundant evidence also supporting a single pathway where CENP-C can be found upstream of CENP-H and CENP-T assemblies [34–40]. In this case, CENP-T/W forms associations with CENP-H/I/K/M, which are both dependent on CENP-C for their kinetochore localization [36,40]. These differences could reflect different experimental approaches, but it is also possible that DT40 chicken cells may exhibit differences in their ability to recruit outer kinetochore components when compared with other systems.

Reproducible differences in the kinetochore-associated proteome were also observed when we compared rapid AID-CENP-T degradation with the more gradual loss of CENP-T/W in the conventional CENP-T^OFF^ and CENP-W^OFF^ KO cells. A number of kinetochore proteins were reduced on chromosomes isolated from conventional CENP-T^OFF^ and CENP-W^OFF^ cells. These included components of the RZZ complex, Spindly, Mad1/Mad2, CENP-E and SKAP (figure 6*b*). SKAP is an interactor of CENP-E that is required for chromosome alignment on the metaphase plate [41]. These reductions were statistically significant for CENP-W^OFF^ chromosomes, but not for chromosomes isolated from the AID-CENP-T:CENP-T^OFF^ cells (figure 7). In CENP-W^OFF^ cells members of the CCAN, including CENP-I, -K, -N, -Q, -R and -P, were also reduced. By contrast, components of the proteasome degradation machinery were significantly increased on mitotic chromosomes isolated from the AID-CENP-T:CENP-T^OFF^ cells (figure 7, white circles in the upper left quadrant). This was not seen for the two conventional KO cell lines.

Interestingly, CENP-O/P/Q/R were not depleted from conventional CENP-T^OFF^ chromosomes, but they were partially depleted from CENP-W^OFF^ chromosomes (figure 6*b*). This difference may reflect the fact that chromosomes isolated from CENP-T^OFF^ cells still had approximately 50% of CENP-T/W/S/X remaining, whereas levels of the complex were much lower in chromosomes isolated from CENP-W^OFF^ cells. It is possible that CENP-O/P/Q/R association with chromosomes might require a threshold level of CENP-T/W/S/X.

Together, these results suggest that the time scale of removal of CENP-T/W from kinetochores may influence the composition of the resulting kinetochores.

## 3. Discussion

Here, we have used two different conditional knockout protocols to distinguish the roles of CENP-T/W in kinetochore assembly during the cell cycle from its roles in the maintenance of kinetochore structure during mitosis.

In conventional conditional knockouts, doxycycline addition silences transcription of the rescuing cDNA in CENP-T^ON/OFF^ and CENP-W^ON/OFF^ cells and target protein levels fall gradually over the subsequent 2–3 days. During this time, cells traverse several cell cycles (the doubling time of DT40 cells is approx. 9 h). Thus the proteome of mitotic chromosomes isolated at the end of the experiment reflects the results of assembling kinetochores in the presence of decreasing levels of CENP-T/W.

By contrast, using the auxin degron system [12] AID-CENP-T is depleted from mitotic cultures in an hour or less. In this experiment, cells enter mitosis with normal kinetochores, but then lose CENP-T over a 2 h period. Changes in the proteome thus reflect the loss of components from pre-assembled kinetochores when CENP-T/W is removed.

The fact that CENP-T is a core component of a macromolecular complex does not affect its ability to be degraded from kinetochores using the AID system. AID-CENP-T degradation is fast and efficient, particularly during mitosis, when the protein pool is not replenished by translation. This rapid stripping of CENP-T/W inactivates the kinetochore and greater than 70% of cells halt in mitosis after only 12 h using the AID system. Our data reveal a progression from a ‘normal’ prometaphase arrest into phenotypes characterized by hypercondensed chromosomes displaced from abnormal spindles. Interestingly, some apparent lateral associations of chromosomes with microtubules were observed by CLEM. Similar observations were also reported following rapid kinetochore disruption following the microinjection of anti-centromere antibodies [42], and through live cell imaging of CENP-W^OFF^ and Ndc80^OFF^ conditional KO cells [6,43].

Following gradual depletion of CENP-T/W, the levels of chromosome-associated CENP-A and CENP-C remain unchanged. This supports the viewpoint that CENP-A and CENP-C assembly at centromeres is independent of CENP-T/W [1,6,28,31]. By contrast, there was a slight decrease in chromosomal levels of several other CCAN members, including CENP-I, CENP-K, CENP-N, CENP-Q, CENP-P and CENP-R. Loss of CENP-T/W during kinetochore assembly also resulted in lower levels of the KMN network, the RZZ complex, Spindly, Mad1/Mad2, CENP-E and SKAP on mitotic chromosomes. BubR1 and the Ndc80 complex have been previously associated with CENP-E kinetochore localization [44–47] and Mad1 depletion does not abolish CENP-E in human cells [48] or Mad1 immunodepleted egg extracts [49]. More recently, CENP-E recruitment to unattached kinetochores has been shown to be dependent on Mad1. In fission yeast, Mad1 can bind Cut7 (Kinesin motor) to unattached kinetochores. An equivalent interaction between human Mad1 and CENP-E was also shown [50].

Recent studies in budding yeast have revealed an association between KNL-1 and Mad1 that is mediated by Bub1 [51]. A Mad1:Bub1 interaction in *C. elegans* is important for targeting Mad1/Mad2 to kinetochores [52]. It has also been suggested that Bub1-RZZ complex-Mad1/2 is docked to KNL-1 via Bub1–Bub3 interactions [53]. Under all conditions tested here, Bub1 levels on chromosomes did not change in CENP-T/W/S/X depleted cells, even when KNL-1 was significantly depleted. This suggests an alternative pathway independent of Bub1 for the recruitment/maintenance of Mad1 at kinetochores in nocodazole-treated DT40 cells.

Mad1 kinetochore localization is also dependent on the RZZ complex [54–56], and interactions between these components in *Drosophila* embryos has recently been shown through co-immunoprecipitation experiments [57]. This complex in turn requires KNL-1 for kinetochore targeting [56,58], although loss of KNL-1 (or Bub1) in mitotically arrested human cells does not completely abolish Mad1 or RZZ kinetochore localization and implicates an additional mechanism for complete recruitment [59]. Interestingly, the CENP-T stripping from chromosomes during mitosis led to a significant downstream depletion of KNL-1, but changes in RZZ/Spindly/Mad1/Mad2/CENP-E abundance on chromosomes were not observed. Possibly, once they are assembled, other interactions/pathways may be involved in the association of this complex with mitotic kinetochores.

An independent proteomic study of conventional knockouts of eleven kinetochore proteins and assembly factors has strongly suggested the existence of a super-complex that we refer to as the RZZ-MES (Rod, Zw10, zwilch, Mad1, CENP-E and Spindly [11]). The present data support the hypothesis that CENP-E exhibits functional links with RZZ/Spindly/Mad1/Mad2 in mitotic chromosomes. It is interesting that this putative super-complex requires CENP-T/W association with chromosomes during kinetochore assembly in order for optimal stable association with mitotic chromosomes, but is independent of CENP-T/W once kinetochores have been assembled.

Stripping of CENP-T/W from mitotic kinetochores had no effect on the levels of CENP-A, CENP-C or any other CCAN components on isolated chromosomes. It did, however, result in a partial loss of KMN components, confirming a previous study looking at Ndc80 levels after CENP-T degradation by the AID system [19]. It is possible that the KMN proteins remaining after CENP-T stripping are retained via interactions with CENP-C. The N terminus of CENP-C is important for outer kinetochore assembly as established by ectopic localization experiments [28,29,31,32,39] and interacts with Mis12 components [28–30], possibly providing a second platform for Ndc80 assembly [19,32]. In budding yeast, the Mtw1 complex (Mis12 complex) has been shown to associate with Ndc80 components and this interaction is mutually exclusive of Cnn1 (CENP-T homolog)-Ndc80 direct binding [60–62].

### 3.1. Perspectives

Most strategies for conditional knockouts of essential genes involve either blocking transcription of the target gene or inactivating the mature mRNA, with the result that the target protein is depleted from cells over the course of several cell cycles [63]. The availability of the AID system allows us to direct the destruction of a target protein within an hour or two—certainly within the normal span of a single vertebrate cell cycle. This allows us to examine the differences between (i) gradually inactivating a protein and thus interfering with the assembly of downstream complexes, and (ii) allowing the assembly of those complexes, and then rapidly stripping them of the target protein. For CENP-T/W both strategies inactivate the kinetochore. Interestingly, interfering with kinetochore assembly inhibits the ability of the putative RZZ-MES super-complex (as well as Mad2) to stably associate with mitotic chromosomes, whereas this association, once formed, is stable to CENP-T/W-dependent stripping of the complex. Rapid removal of CENP-T/W also appears to selectively inhibit the ability of kinetochores to make end-on attachments with microtubules. In the future, it will be interesting to use this approach to differentiate the roles of other subcomplexes in kinetochore assembly versus structural maintenance during mitosis.

## 4. Material and methods

### 4.1. Cell culture, drug treatments and transfections

DT40 cell lines were cultured in RPMI media (Invitrogen) with 10% (v/v) heat inactivated FBS, 1% chicken serum (Gibco) and 1% penicillin/streptomycin at 39°C. CENP-T^ON/OFF^ and CENP-W^ON/OFF^ conditional tetracycline-repressive KO cell lines [6] were treated with 0.2 µg ml^−1^ doxycycline (BD Biosciences) to silence the transgene. A 12-h treatment with 0.5 µg ml^−1^ nocodazole (Sigma) was used to block DT40 cells in mitosis. The AID-CENP-T:CENP-T^ON/OFF^ cell line was maintained in 0.5 µg ml^−1^ doxycycline at all times. One hundred and twenty-five microlitres of auxin (3-indolylacetic acid; Fluka analytical) was diluted in chemical grade ethanol and used at 125 µm to promote the degradation of AID-tagged CENP-T by the proteasome.

The AID-CENP-T stable cell line was generated using electroporation. Cells were resuspended in ice-cold Optimem at a dilution of 1–2 × 10^7^ cells ml^−1^. Approximately 0.5 × 10^7^ cells were distributed into a cuvette (BioRad; 0.4 cm) with 8–10 µg of the plasmid DNA and placed on ice for 5–10 min. To generate the OsTIR1-T2A-AID-GgCENP-T vector, the OsTIR1-T2A-AID sequence was digested from a pUC57 vector and inserted N-terminal to the GgCENP-T ORF sequence within a pN1 plasmid [6]. T2A sequences differ from internal ribosome entry site (IRES) sequences in that the two polypeptides arise due to ribosomal skipping rather than translation re-initiation [64–66]. As a result, they are produced in nearly equal amounts [67–69]. Cells were electroporated at 300 mA and 950 µF (Gene Pulser Xcell Electroporation System, BioRad) and maintained on ice for a further 5 min. Transfected cells were selected with Geneticin (1.5 mg ml^−1^; Gibco) and Zeocin (400 µg ml^−1^; Invitrogen), and positive clones identified by Western blot analysis.

The GFP-GgCENP-A construct was generated by cloning GgCENP-A into the pEGFPC1 vector (Clontech) with a 17-amino acid linker [70]. The Neon transfection system (100 µl kit; Invitrogen) was used to transiently transfect GFP-CENP-A into DT40 cells according to the manufacturer's instructions. Electroporation protocol was set to a voltage of 1700 V, width 20 ms and pulse 1. After electroporation, cells were resuspended in pre-warmed medium (without antibiotics). For experiments with the AID-CENP-T:CENP-T^ON/OFF^ cell line doxycycline was added 2–3 h after electroporation.

### 4.2. Cell assays

Viable cells were scored using trypan blue (Sigma). The Annexin V-PE-Cy5 Apoptosis detection kit (Biovision) was used according to the manufacturer's instructions. Flow cytometry was used to detect positive cells using a FACsCalibur flow cytometer and CellQuest Software (BD Biosciences).

### 4.3. SILAC labelling, chromosome isolation and LC-MS/MS analysis

DT40 cells were cultured in RPMI media (Invitrogen) with 10% (v/v) dialyzed FBS (Sigma), 100 µg ml^−1^ U-^13^C_6_^15^N_2_-l-lysine:2HCl and 30 µg ml^−1^ U-^13^C_6_^15^N_4_-l-arginine:HCl (Sigma) for five to six cell cycles. To block in mitosis, cells were treated with 0.5 µg ml^−1^ nocodazole (Sigma) for 12–13 h giving a mitotic index between 80 and 95%. Equal numbers of differentially labelled cells blocked in mitosis were mixed and the chromosomes isolated [71,72]. An average of three chromosome isolates were combined for each single biological replicate.

Denaturing protein gel electrophoresis was carried out using NuPAGE Bis-Tris 3–12% gels and MOPS SDS running buffer (Invitrogen). The gel was stained with Imperial protein stain (Thermo Scientific) and In-gel digestion with trypsin performed as described previously [73]. Peptides were separated into 28–48 different fractions (depending on the original protein concentration of the sample) using an Ultimate 3000 HPLC system (Dionex) using a PolySULFOETHYL A SCX column (200 × 2.1 mm, 5 µm particles, 200 A pores, PolyLC Inc). Salt gradients of Buffer A (5 mM dipotassium hydrogen phosphate, 10% ACN adjusted to pH 3 using phosphoric acid) and Buffer B (5 mM dipotassium hydrogen phosphate, 10% ACN, 1 M potassium chloride adjusted to pH 3 using phosphoric acid) were used to separate peptides at a flow rate of 200 µl min^−1^. After SCX fractionation the peptide solutions were mixed 1 : 1 with 0.1% trifluoroacetic acid. Desalting was performed using C18-StageTips [74,75].

The LC-MS/MS analysis was performed on an LTQ-Orbitrap Velos (Thermo Fisher Scientific) using a Waters nano Acuity HPLC system with a nanoelectrospray ion source. Depending on the amount of peptides for each fraction, gradients were run for between 2 and 5 h before analysis. The LTQ-Orbitrap Velos was operated in the data-dependent mode with up to 20 MS MS^−1^ scans recorded for each precursor ion scan.

Quantitation of SILAC Pairs was performed using MaxQuant v. 1.3.0.5 [76] and the peptide search engine Andromeda [77] using a Chicken UniProtKB database with reviewed and unreviewed sequences (downloaded July 2013). Additional kinetochore sequences that needed to be input separately included Aurora B, Ska1, Spc24, HP1alpha, predicted CASC5: gill363734068 and predicted: E3 SUMO protein ligase RanBP2-like: gill363729033. For analysis, the enzyme was set to Trypsin/P with a maximum of two missed cleavages. The mass tolerance for the first search and main search were set to 20 and 6 ppm, respectively. Carbamidomethylation of cysteine was set as a fixed modification, while oxidation of methionine and protein N-acetylation were defined as variable modifications. A minimum of 1 razor or unique peptide and a false-discovery rate (FDR) of 1% were required for protein identification.

For a protein to have quantifiable readings, SILAC ratios must have been generated using at least 1 unique peptide pair and represented in both biological replicates. SILAC outputs from MaxQuant were normalized to Histone H4 ratios. Histone H4 is highly abundant on chromosomes and can be used to account for unequal mixing of cells in the early stages of each experiment [72].

Special procedures were used for CENP-X, which was not detected in one repeat and only quantitated with 1 H/L ratio count under AID-CENP-T SILAC conditions using MaxQuant. Because CENP-X is a small protein (80 amino acids) formed mostly from a histone-fold like domain [7,8], it has a low number of unique peptide sequences available for quantitation. To validate H/L ratios in AID-CENP-T SILAC conditions, we identified a unique CENP-X peptide that was successfully used for quantitation across all other samples. We then manually determined peak areas of an extracted chromatogram from the heavy and light precursor ion measured using Skyline software (electronic supplementary material, figure S2*a*–*c*) [22,23]. Thus, we were able to reliably quantify CENP-X in all samples.

### 4.4. Statistical analysis of SILAC data

R (www.r-project.org) and RStudio (www.rstudio.com) were applied for analysis and the generation of graphical figures. Statistical analysis was performed using the limma package in R/Bioconductor [24–27]. Proteins with an adjusted *p* < 0.05 (representing an FDR of 5%) and that had H/L SILAC ratios greater than 1.5-fold-change were considered to be differentially abundant on chromosomes.

### 4.5. SDS-PAGE, gel staining and immunoblotting

Proteins were sonicated, boiled in sample buffer (1% SDS, 16.7 mM Tris–HCl pH 6.8, 5% sucrose, 0.67 mM EDTA, 10% B-Mercaptoethanol (v/v)) and resolved using SDS-PAGE [78] with 10–15% polyacrylamide gels and 8 M urea in the stacking gel for better resolution of chromosomal proteins (electrophoresis apparatus; BioRad). For gel staining, protein bands were visualized using InstantBlue (Expedeon). For immunoblotting, proteins were transferred to nitrocellulose membranes and blocked with 5% non-fat milk in PBS-0.1% Tween for 1–2 h.

Primary antibodies used for immunoblotting included rabbit anti-GgCENP-T (1 : 2000) [6], rabbit anti-GgNdc80 (1 : 2000) [43], mouse anti-GgINCENP (1 : 3) [79], mouse anti-Histone H3 (1 : 500; Abcam), rabbit anti-GgTopo IIalpha (1 : 500) [80], mouse anti-alpha Tubulin (1 : 2000; B512 Sigma) and anti-GgCyclin B2 [81]. When using the LI-COR Odyssey system, membranes probed with secondary antibodies (IRDye 800 CW/IRDye 680; LI-COR Biosciences) were washed for at least 45 min with 0.1% Tween in PBS and a final wash performed with PBS for 5 min. Median fluorescence intensities for individual protein bands were subsequently determined using a CCD scanner (Odyssey; LI-COR Biosciences).

### 4.6. Immunofluorescence microscopy

DT40 cells adhered to coverslips using 0.1% poly-l-lysine (PLL; Sigma) solution were washed for 2 min with pre-warmed PBS and fixed for 8 min with 4% paraformaldehyde at room temperature. This was followed by PBS washes, incubation with 0.15% Triton X-100/PBS for 2 min and a final wash with PBS. A block using 1% bovine serum albumin (BSA) diluted in PBS for 30 min at 37°C was performed. Rabbit anti-GgCENP-T (1 : 1000) [6] in blocking solution was incubated for 1 h and washed three times with 0.15% Tween/PBS. This was followed by incubation for 1 h with fluorophore-conjugated secondary antibodies (Alexa Flour 594; Jackson ImmunoResearch Laboratories, Inc.) and additional wash steps. Coverslips were mounted on slides using VectorShield containing DAPI (Vector Labs).

Images were taken with a Wide Field Deconvolution Microscope (DeltaVision Core system, Applied Precision), based on an Inverted Olympus IX-71 microscope stand with Olympus UPlanSApo 100 × oil immersion objective (NA 1.4), a 250 W Xenon light source and camera (CoolSnap HQ, Photometrix). Shutter and stage were controlled through SoftWorx (Applied Precision). Z sections were deconvolved using the constrained iterative algorithm on SoftWorx [82,83]. Max intensity projections were created either using SoftWorx or ImageJ. To make comparisons and quantitative measurements images were processed in ImageJ by thresholding the maximum and minimum intensities for three-dimensional projections. The default setting for CraQcode v. 1.06 [84] were modified and used to generate quantifiable measurements. GFP-CENP-A signals were used to locate kinetochores and the intensity of the Gg-CENP-T signal measured at these points.

### 4.7. Chromosome spreads

After drug treatments (concentrations described previously and outlined in the electronic supplementary material, figure S3*b*), mitotic cells were counted and resuspended in warm 75 mM KCl to a concentration of 60 × 10^4^ cells ml^−1^. After a 7 min incubation, 100 µl of cell suspension was loaded into a cytofunnel and centrifuged at 1800 r.p.m. for 10 min onto a glass slide (Shandon Cytospin 4). Slides were immersed in KCM buffer (10 mM Tris–HCl pH 8.0, 120 mM KCl, 20 mM NaCl, 0.5 mM EDTA, 0.1% v/v Triton X-100) for 10 min at room temperature and then carefully dried. Samples were blocked using 100 µl of 1% BSA in KCM buffer for 1 h and incubated for a further hour with primary antibodies (anti-GgCENP-A; 1 : 500, anti-GgCENP-T; 1 : 1000). After washing the slide with KCM buffer a secondary antibody incubation was performed for 45 min (anti-chicken Alexafluor488; 1 : 500). This was followed by additional washes, fixation with 4% formaldehyde (diluted in KCM buffer) for 10 min and a final incubation with DAPI in PBS for 5 min. Washes with ddH_2_O were performed before mounting with a coverslip.

Images were taken as described in the above section and analysed in ImageJ. For quantitative measurements, chromosomes with clear constriction sites were identified via DAPI channel images. From here, a circle with a defined area was used to find the mean fluorescence intensity of CENP-T or CENP-A signals at constriction sites/centromeric regions. The minimum signal value was used as local background and subtracted from each chromosomal reading.

### 4.8. Electron microscopy

Cell suspension containing 1.6 × 10^5^ DT40 cells were seeded onto PLL coated gridded dishes (MatTek, USA) and left to adhere for 30 min before fixation. The CLEM processing method was adapted from an established protocol [85]. Briefly, cells fixed for 1 h with 3% glutaraldehyde and 0.5% paraformaldehyde in 0.2 M cacodylate buffer containing 5 µg ml^−1^ Hoechst were washed with PBS, mitotic cells of interest were identified using the DeltaVision Core system (Applied Precision) and the cell positions mapped using the etched coordinates on the gridded dishes. Samples were then processed as described previously [85]. Micrographs were acquired using a Philips CM120 transmission electron microscope (FEI) and Gatan Orius CCD camera (Gatan).

## Supplementary Material

Supplementary Figure 1. Reproducibility between proteomics data sets.

## Supplementary Material

Supplementary Figure 2. CENP-X SILAC ratio in AID-CENP-T SILAC samples.

## Supplementary Material

Supplementary Figure 3. Verification of the CENP-W and CENP-T conditional KO cell lines.

## Supplementary Material

Supplementary Table 1. Kinetochore list.

## Supplementary Material

Supplementary Table 2. Significantly depleted chromosomal proteins.
